# Fungi, Drugs, and Money: New Issues, Old Problems

**DOI:** 10.1100/tsw.2001.46

**Published:** 2001-04-27

## Abstract

Nature leads this week with a story about a modern potato famine - this time in Russia. Science explores the continuing skirmishes over drug-patent profits.

*Phytopthora infestans*, a virulent potato fungus that originated in Mexico, is beginning what could become a massive assault on Russian potato crops, reports *Nature*. Researchers at Cornell University, led by plant scientist Kandukuri Raman, observe that much like nineteenth century Irish farmers, poor agricultural Russians depend on potatoes for their basic subsistence and that the blight could result in famine. The potato late blight, which becomes more deadly to crops through rapid adaptation in subsequent fungal generations, also creates spores that survive in the ground throughout frigid Russian winters to infest new potatoes in the summer. The Cornell Eastern Europe-Mexico (CEEM) project in cooperation with the U.S. Department of Agriculture plans to give farmers a one-up by providing blight-resistant seed, but is limited in resources, says *Nature*. Fungicides capable of defeating the potato bug are not accessible for potato growers, probably due to their cost. Scientists at the N. I. Vavilov All-Russian Scientific Research Institute of Plant Industry in St. Petersburg have teamed with CEEM and other researchers to investigate the molecular interactions between *P. infestans* and the vegetables. The teams also hope to find wild potato species that may be resistant to the disease, since formerly hardy crop types have failed to keep pace with the blight's deadly progress. *Science* did not cover this story.

The University of Minnesota may stand as an unlikely gladiator in drug-patent profit battles, reports *Science* in its lead story. Just two years after winning a patent dispute with Glaxo Wellcome on behalf of university researchers over the HIV drug, abacavir, the school is again put into the arena. Students last month staged a demonstration to pressure administrators to not enforce the patent on Ziagen, the brand form of abacavir, in Sub-Saharan Africa. University official Christine Mazair reportedly told *Science* that the university will back drug giant GlaxoSmithKline, with whom the school shares the patent, when the corporation implements its plan to lower HIV drug prices in AIDS-stricken regions of Africa. The university will not give up its stake in the patent revenues altogether, however, said Mazair. Last December, the U.S. Senate compelled the National Institutes of Health into the ring by asking that the agency keep track of publicly funded drug discoveries that lead to huge revenues. Next, the NIH was asked to develop a plan to recapture some of the profits. The international debate encapsulated in these developments could have far-reaching impact on how drugs are discovered and commercialized by university-industry-government triangles. Interestingly, no participants in these battles have noted the possible time-to-market lag that deeper government controls would most likely create. The extra paperwork alone of the NIH as a new participating agency in the complex process of getting new pharmaceuticals to the market could add serious time to the lab-to-treatment equation.
